# Seminal fluid proteins induce transcriptome changes in the *Aedes aegypti* female lower reproductive tract

**DOI:** 10.1186/s12864-021-08201-0

**Published:** 2021-12-15

**Authors:** I. Alexandra Amaro, Yasir H. Ahmed-Braimah, Garrett P. League, Sylvie A. Pitcher, Frank W. Avila, Priscilla C. Cruz, Laura C. Harrington, Mariana F. Wolfner

**Affiliations:** 1 Department of Entomology, Cornell University, Ithaca, NY 14853 USA; 2 Department of Biology, Syracuse University, Syracuse, NY 13244 USA; 3 Max Planck Tandem Group in Mosquito Reproductive Biology, Universidad de Antioquia, Medellín, 050010 Colombia; 4 Department of Molecular Biology and Genetics, Cornell University, Ithaca, NY 14853 USA

**Keywords:** *Aedes aegypti*, Seminal fluid proteins, RNA-Seq, Post-mating responses, Immunity, Proteolysis, Transcriptional control

## Abstract

**Background:**

Mating induces behavioral and physiological changes in the arbovirus vector *Aedes aegypti*, including stimulation of egg development and oviposition, increased survival, and reluctance to re-mate with subsequent males. Transferred seminal fluid proteins and peptides derived from the male accessory glands induce these changes, though the mechanism by which they do this is not known.

**Results:**

To determine transcriptome changes induced by seminal proteins, we injected extract from male accessory glands and seminal vesicles (MAG extract) into females and examined female lower reproductive tract (LRT) transcriptomes 24 h later, relative to non-injected controls. MAG extract induced 87 transcript-level changes, 31 of which were also seen in a previous study of the LRT 24 h after a natural mating, including 15 genes with transcript-level changes similarly observed in the spermathecae of mated females. The differentially-regulated genes are involved in diverse molecular processes, including immunity, proteolysis, neuronal function, transcription control, or contain predicted small-molecule binding and transport domains.

**Conclusions:**

Our results reveal that seminal fluid proteins, specifically, can induce gene expression responses after mating and identify gene targets to further investigate for roles in post-mating responses and potential use in vector control.

**Supplementary Information:**

The online version contains supplementary material available at 10.1186/s12864-021-08201-0.

## Introduction

*Aedes aegypti* is a significant mosquito vector of the viruses that cause Zika (ZIKV), chikungunya (CHIKV), yellow fever (YFV), and dengue (DENV) [1–4]. There are no approved anti-viral therapies for *Aedes*-borne pathogens [5, 6] and while an effective vaccine for yellow fever exists, supplies have become depleted in recent outbreaks [7]. A commercially licensed vaccine for dengue has limited efficacy and is not widely used [8]. Efforts to reduce disease burden rely on vector control. However, conventional mosquito control measures using insecticides can be operationally difficult and ineffective in areas where insecticide resistance is a major problem [9], and may have detrimental effects on beneficial insects [10]. Population replacement or reduction control strategies that involve deployment of modified mosquitoes show promise for control [11]. However, these approaches rely on successful mating and reproduction [12].

Mating in many insects, including *Ae. aegypti*, initiates behavioral and physiological changes in females (reviewed in [13]). Seminal fluid proteins (SFPs), which are primarily produced in the male accessory glands (MAG) as well as the ejaculatory duct, are transferred to females during mating and play a large role in inducing post-mating responses [13–15]. Much of the detailed work on the nature, function, and effects of SFPs has been conducted in *Drosophila melanogaster* (reviewed in [13]). While less is known about *Aedes* male accessory gland-derived SFPs, a recent analysis cataloged 280 SFPs in *Ae. aegypti* [16], similar to the number reported for *D. melanogaster* [17]. However, specific functionality for nearly all of these proteins has not been determined. Mating or injection of SFP-containing MAG extracts stimulates *Ae. aegypti* females to oviposit developed eggs several days after blood meal ingestion [18–22]. In addition, survival of mated and MAG extract-injected females is higher than virgins' irrespective of whether a blood meal was consumed [22], and females that mate with non-SFP-depleted virgin males survive longer [23]. Females are monandrous and demonstrate reduced mating receptivity after an initial mating [24, 25]. This effect sets in quickly, with only a quarter of females being receptive to re-mating within 2 h after the first mating event, and complete mating refractoriness is established within 24 h post-mating [26]. A low dose of MAG extract is sufficient to induce this effect [25, 27], which persists through multiple gonotrophic cycles [26]. While the transfer of male mature Head Peptide-1 contributes to short-term transient refractoriness [28], the male proteins/peptides responsible for long term establishment over the duration of the female lifespan are unknown. MAG extract injection into a female, or mating, reduces the likelihood that the female and her potential mate will harmonize their flight tones [29], a mechanism by which SFPs could reinforce resistance to re-mating. It is likely that there are additional yet unknown effects of SFPs on female mosquito post-copulatory biology and behavior.

Knowing the molecular pathways that are activated in *Ae. aegypti* females post-mating would be helpful in guiding functional assays and ultimately understanding mosquito post-mating biology and vector control targets. However, these pathways are presently unknown. Transcriptome analysis, first with microarrays and now with Next Generation Sequencing (NGS), has been a valuable tool to examine gene expression changes in whole bodies or specific tissues of mated females in a variety of insect orders, including Diptera (Drosophilidae, Tephritidae, Aleyrodidae, Culicidae), Hymenoptera, Lepidoptera, and Coleoptera [30–45]. We previously examined transcript-level changes in the *Ae. aegypti* female lower reproductive tract (LRT, which consists of the bursa, spermathecae, and oviducts) at 0, 6, and 24 h after mating and identified transcriptional changes in protease and antimicrobial peptide genes as well as molecules that regulate immunity [46]. Similar analysis of various reproductive tissues minus the ovaries in *Anopheles gambiae* and *D. melanogaster* have identified changes in transcripts associated with metabolic function, catalytic activity and immunity [47–50]. Identification of a cluster of genes that respond to mating in *An. gambiae*, all linked by 20E hormonal regulation, led to the discovery that 20E was an activator of post-mating responses in that mosquito [49]. This work demonstrated the power of transcriptome analysis to provide insights into female reproductive pathways.

Specific female post-mating transcription changes are induced by different ejaculate components, as demonstrated in *D. melanogaster* where different sets of female genes are regulated by sperm, by SFPs or by the act of, or some other component of mating [31, 33]. Transcript profiles of *An. gambiae* and *Ceratitis capitata* females after mating to sperm-less vs. wild-type males gave insights into sperm-specific female responses [51–53]. However, it is not known which genes in the *Ae. aegypti* female LRT respond specifically to seminal fluid proteins, the initiators of post-mating responses [13]. Identification of this subset of genes from among those in females that respond to mating will provide insight into the as-yet unknown mechanisms that underlie post-mating responses. In *D. melanogaster*, SFPs transit into the hemolymph through damage to the female vaginal wall during copulation [54, 55] and injection of SFPs into *Drosophila* hemolymph causes post-mating-like changes [56]. As the injection of MAG extracts, but not saline, into *Ae. aegypti* virgin females also causes post-mating-like changes [22, 25, 27, 29], we utilized this means to introduce seminal fluid proteins into females to investigate their influence on transcript levels. Transcripts thus identified are regulated either directly or indirectly upon introduction of seminal proteins during a natural mating and identify female pathways regulated by SFPs that participate in post-mating responses.

## Results and discussion

### Saline injection into *Ae. aegypti* females induces expression of genes involved in wounding and immunity pathways in their LRTs

**Fig. 1:**
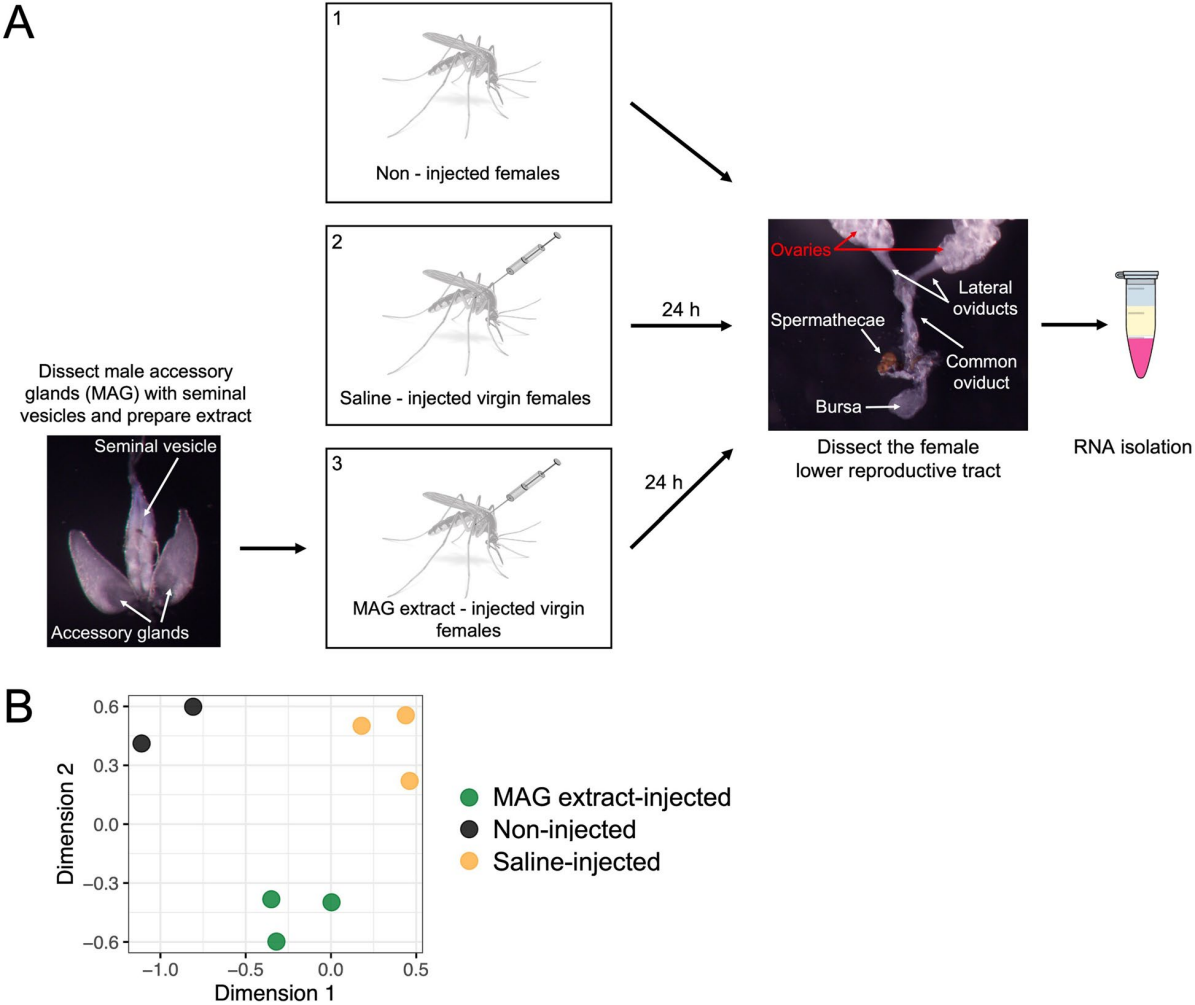
Study design methods from dissection to RNA isolation and data set analysis. (**A**) The lower reproductive tract (tissues with white text except for the ovaries labelled in red) was dissected from virgin females treated in one of three experimental conditions 1) no injection. 2) injected with saline 24 h prior to dissection 3) injected with extracts derived from male reproductive tissue (male accessory glands and seminal vesicles) 24 h prior to dissection. Total RNA isolated from these tissues was used in library construction for Illumina sequencing. (**B**) Multi-dimensional scaling plot of samples and replicates from this study

**Fig. 2:**
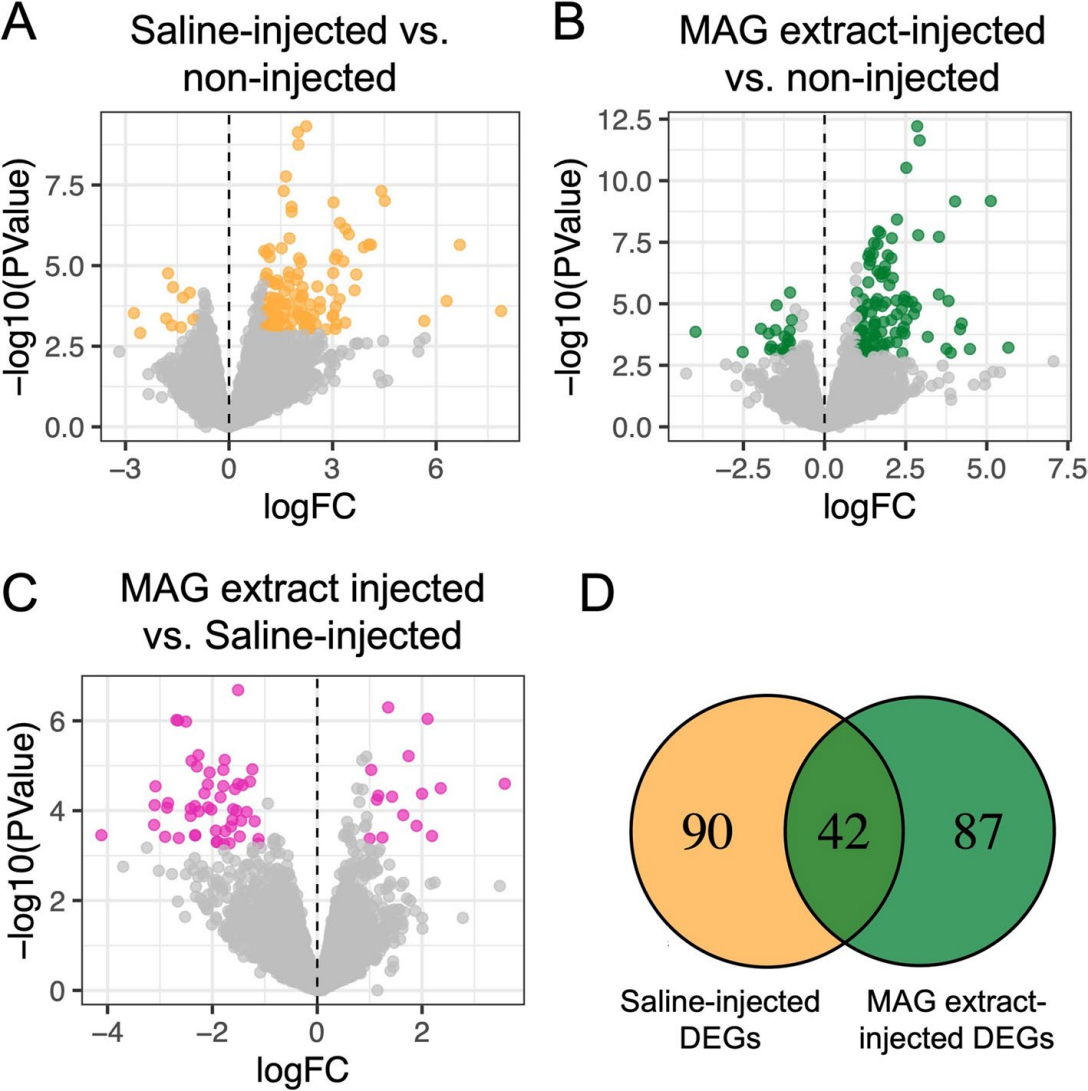
Transcript-level changes observed in female LRTs 24 h after injection. Volcano plots depicting changes in transcript levels in (**A**) saline-injected females or (**B**) MAG extract-injected females each compared to non-injected females. (**C**) Transcript level comparisons between MAG extract-injected and saline-injected females are similarly depicted. Significant transcript level changes are highlighted (*P* < 0.05; FDR < 0.05) in yellow, green, and pink. (**D**) Venn diagram comparing number of DEGs in saline- and MAG extract-injected over non-injected mosquitoes

The female mosquito’s lower reproductive tract (LRT) is critical to sperm storage, egg transit, and fertilization. These organs undergo transcriptome changes after mating [46, 57], presumably as part of the female’s transition to high reproductive efficiency. To determine which of these changes are induced by seminal fluid proteins (SFPs), we examined transcriptome changes in the LRT at 24 h, the time at which the greatest number of transcriptional changes were observed in the female LRTs of mated *Ae. aegypti* [46] and *An. gambiae* [49], following injection of lysate derived from the male accessory gland (MAG), the primary tissue that produces SFPs, and associated seminal vesicles, hereafter referred to collectively as MAG extract (Fig. 1A). To identify transcriptome changes that were induced simply by needle-wounding and saline, we first compared the transcriptomes of the LRTs of saline-injected females with those of non-injected females. We observed differentially expressed genes (DEGs) at 24 h after saline injection, consisting of 10 down-regulated and 122 up-regulated genes. (Fig. 2A, Supplementary Table 1). As expected for a response to injection, DEGs with known or potential function in wounding cascades or immunity pathways in mosquitoes and other insects were represented [58, 59]. These include up-regulation of RNAs encoding peroxidases (AAEL026038, AAEL004388, AAEL004386) and a dopachrome-converting enzyme (AAEL000064) that is involved in melanization pathways [60, 61]. We observed up-regulation of RNAs encoding known or predicted serine protease domains (AAEL002686, AAEL007102, AAEL015533, AAEL015432, AAEL005748, AAEL002624, AAEL006576, AAEL024784), including CLIP-serine proteases (AAEL010773, AAEL002124, AAEL000037, AAEL024669, AAEL002585), which together with a protein containing a serpin domain (AAEL020823) are classes of proteins known to have regulatory control over melanization, coagulation, and immune pathways [58, 62–65]. Additional up-regulated DEGs associated with the immune responses included those with C-Type lectin domains (AAEL011408, AAEL023353), a C-Type lysozyme (AAEL017132) and anti-microbial peptides (AMPs) Defensin C (AAEL003832), Defensin D (AAEL003857), and Holotricin (AAEL017536) [66]. The most up-regulated gene, AAEL008098/PIWI, is involved in RNA silencing as a part of the mosquito anti-viral response [67]. These changes in levels of transcripts with immune function in the LRT indicate a response to wounding from injection and introduction of saline into the thorax.

These results are consistent with other studies that have reported immune activation after injection either alone or with media, confirming the quality of our data [68–72]. Priming *Ae. aegypti* immunity by needle injection with RPMI media leads to increases in relative expression of RNAs encoding the AMPs Defensin, as we also observed for the LRT, as well as Cecropin and Attacin [68]. Circulating hemocytes, immune cells in the hemolymph, that are isolated from *Ae. aegypti* adults following injection of saline show significantly increased protein concentrations when compared to hemocytes from non-injected adults [69], which may reflect activation as hemocytes are recruited to the site of injury. Differential expression in *D. melanogaster* of immune-related, cell proliferation, and cell migration genes is observed in third instar larvae hemocytes following needle puncture [70]. The injection set-up in our study does not lend itself to practically sterilize each needle, hence some of the observed DEGs could be in response to bacteria or other pathogens that were introduced either on the needle or by opportunistic entry into the wound site from the mosquito cuticle. In *D. melanogaster*, activation of cellular immune responses can be achieved by sterile wounding alone [71]. Clot formation due to wounding is not fully understood in mosquitoes, but electron microscopy of sterile needle damage in *Armigeres subalbatus* larvae demonstrates involvement of granulocytes and deposition of melanin [72], which is synthesized by peroxidases and dopachrome-converting enzyme under the regulatory control of serine proteases, each of which was observed to be up-regulated upon injection in our data. Injection into adults is not without consequence, and our dataset revealed these changes in an unbiased manner to include more than the best characterized immune and wounding responses. Seven of the ten down-regulated genes and 67 of the 122 up-regulated genes are currently uncharacterized in the current genome annotation and may define new players in responses to wounding or infection.

### MAG extracts induce genes in the LRT that are involved in a variety of molecular processes

MAG extract injection down-regulated 21 genes and up-regulated 108 genes relative to non-injected females after 24 h (Fig. 2B). To focus our analysis on genes regulated specifically by MAG contents, and not from the injection itself, we removed the 42 DEGs that were also observed in saline-injected females compared to non-injected from our 129 MAG extract-injected DEGs dataset (Fig. 2D, Supplementary Table 1). The remaining 87 genes (68 up-regulated, 19 down-regulated) show significant transcript-level changes in the LRT following MAG extract injection that are involved in a variety of molecular processes (Supplementary Table 2). We observed DEGs with functions in immunity pathways, including pattern recognition receptors such as C-type lectins (AAEL000543, AAEL014382, AAEL012353, AAEL026265), proteins with a peptidoglycan recognition protein domain (AAEL009474, AAEL019745), a gram-negative binding protein (GNBP) (AAEL007626), and macroglobulin (AAEL001794) which can complex with leucine-rich repeat containing proteins (AAEL001414) [58, 59]. MAG extract injection resulted in up-regulation of AAEL019728, a suppressor of cytokine signaling (SOCS) associated with Janus kinases/Signal transducer and activator of transcription protein (JAK/STAT) pathways [73] and cecropins (AAEL029038, AAEL029044). Transcript levels were also altered for 11 genes with a predicted serine protease domain, which is involved in regulatory control of multiple cellular functions [58, 62, 64, 65]. Among other notable detected changes to transcripts were those in genes with predicted juvenile hormone binding protein domains (AAEL001323, AAEL009927), an odorant binding protein (AAEL006109), five genes with associated neuronal function (AAEL014450, AAEL019604, AAEL003413, AAEL023634, AAEL005945), and three genes involved in transcriptional control (AAEL004097, AAEL003861, AAEL013321) (Supplementary Table 2). In the current genome annotation, ten genes are annotated as non-coding RNAs, and we observed ten DEGs that are uncharacterized for which we could also not predict a conserved domain or find a similar alignment to known genes by BLAST.

### Transcript changes that are observed after both mating and MAG extract injection represent gene pathways that are influenced by seminal fluid proteins

**Fig. 3:**
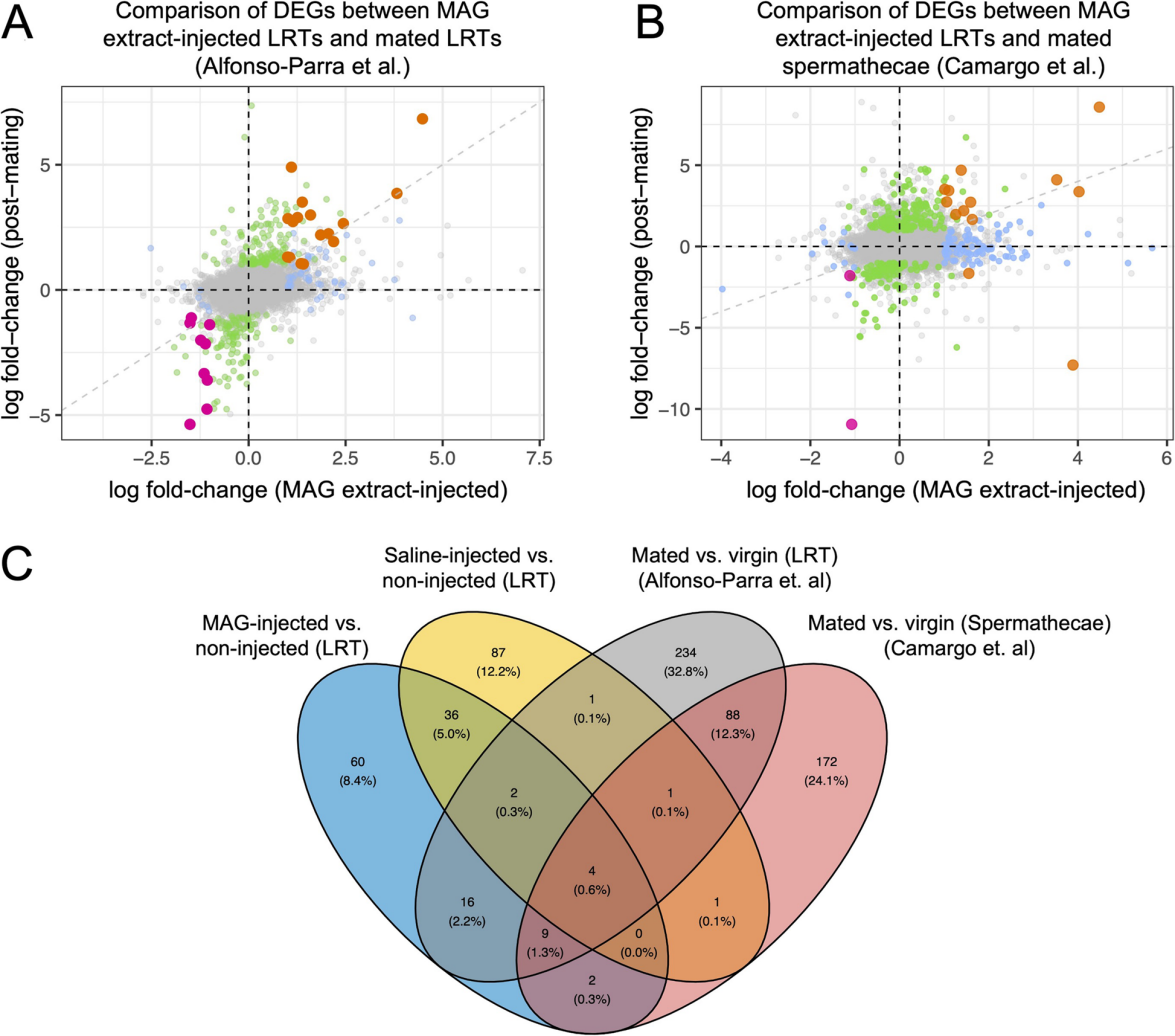
Female reproductive tissues injected with MAG extract or naturally mated after 24 h share DEGs. Comparison between DEGs in the (**A**) LRT [46] or (**B**) spermathecae [57] of mated females (logFC in relation to virgin females on y-axis with significant DEGs noted in green) and LRT of MAG extract-injected females (logFC in relation to non-injected females on x-axis with significant DEGs noted in blue). Dark orange dots denote common up-regulated genes in both studies. Magenta dots denote common down-regulated genes. The dashed diagonal line represents equal log fold-change for genes in the two treatment groups. (**C**) Venn diagram illustrating the DEGs (compared to non-injected or virgins) from four treatment groups

Physical interactions between males and females involved in mating, as well as the transfer of ejaculate comprising of not only sperm, but also SFPs and RNAs all contribute to transcriptional changes in mated females [31, 33, 46]. We previously examined transcriptional changes in response to mating after 24 h in the LRT [46]. To distinguish between transcript changes observed after mating that were due to SFPs, rather than those changes that result from other mating components, we compared DEGs identified after both MAG extract-injection and natural mating. Using an updated analysis of the DEGs at 24 h post-mating with a more recent release of the *Ae. aegypti* genome [74] (Supplementary Fig. 1, Supplementary File 1), we identified DEGs (10 down-regulated and 21 up-regulated) in common between MAG extract-injected and similarly-aged mated females (Fig. 3A, C, Supplementary Tables 2, 3). In particular for the similarly up-regulated genes, there was congruence between the magnitude of the transcript-level changes between the two studies. When the logFC for these genes in each study were plotted together, those genes clustered around the line of equality which represents an equal log fold-change from each study. Futhermore, these commonly altered genes included one down-regulated gene and 5 up-regulated genes that responded to injection of both MAG extracts and saline (Fig. 3A, C, Supplementary Table 3). These 6 genes have functions consistent with immunity and injury pathways, such as defensins and serine proteases, which could explain the presence of these DEGs in saline-injected females. Overall, the 31 overlapping genes are those that are likely to be influenced by SFPs during mating, rather than sperm or mating behavior, and can give insights into important post-mating pathways in females. The DEGs have functions in multiple pathways as discussed below.

#### Transcripts of genes with immune function

We observed changes in LRTs from mated and MAG extract-injected females of RNAs from genes with known or predicted immune function (AAEL000200, AAEL001794, AAEL020192, AAEL007626, AAEL029044, AAEL019728, AAEL003832, AAEL003857) [59]. Expression of these genes was not induced by needle puncture and saline alone, because when we compared transcript levels in MAG extract-injected versus saline-injected LRTs (Fig. 2C, Supplementary Table 2), we found significantly higher transcript levels for AAEL003832/Defensin C (logFC 1.43), AAEL029044/predicted Cecropin (logFC 1.90), and AAEL005641/C-Type lectin (logFC 2.35), indicating that induction of these genes is above what would be seen for injury alone and could reflect additional up-regulation due to SFPs. Additionally, we observe a statistical enrichment of DEGs with Gene Ontology (GO) terms corresponding to immune functions (Supplementary Fig. 2). This enrichment was not observed with DEGs from saline-injected females, indicating that transcriptional changes in a number of immune genes are the result of introduction of MAG extract components, not from injury due to injection. Although the MAG tissue was immediately removed to fresh saline prior to homogenization, males were not surface-sterilized prior to dissection. Thus, we cannot exclude the small possibility that some microbial contaminants from the carcass could be in our extract and contribute at some level to induction of immune response-genes in females. Similar mating-induced changes in immune gene transcript levels have also been reported in mated *Drosophila* females [30, 31, 45, 47, 48, 75–78], including a recent analysis finding distinct mating-induced transcriptional changes in immune genes in the different tissue types that comprise the *D. melanogaster* female reproductive tract [50]. Other insects demonstrate changes in immune gene transcript levels after mating, including *An. gambiae* [49], *Bactrocera tryoni* [41], *Bemisia tabaci* [44], ants [79], and *Bactrocera dorsalis* [39]. The overall significance of differential expression of immune genes after mating is the subject of continued study [80–83].

Recent work has highlighted roles for immune genes in reproductive pathways. For example, C-type lectins, such as AAEL005641/C-Type lectin which was one of the DEGs in our dataset, have been classically associated with immune function, yet in *Ae. aegypti* knockout of C-type lectin GCTL-3 using CRISPR decreased dengue virus susceptibility, but also altered germ line development and reduced fertility [84]. The complement-like molecule Thioester-containing protein 1 (TEP1) in *An. gambiae* recognizes malaria ookinetes and also clears damaged sperm from the testes [85]. While we did not observe the *Ae. aegypti* homolog of TEP1 as a DEG responsive to MAG extract injection, we did observe a macroglobulin/complement gene to be differentially regulated by MAG extract (AAEL001794). It will be interesting to investigate the immune genes influenced by SFPs uncovered here for roles in reproduction, particularly the Suppressor of Cytokine Signaling (SOCS) gene’s involvement in JAK/STAT immune signaling pathways [73].

#### Transcripts encoding predicted proteases

Proteins with serine protease function (AAEL001178, AAEL010867, AAEL014567, AAEL015533, AAEL000037) shared between MAG extract-injected and mated LRTs are involved in a variety of cellular processes, including immunity pathways as discussed previously, but also in reproduction. Serine proteases comprise a large fraction of proteins found in the seminal fluid of both insects and mammals (reviewed in [86]). These proteins contribute to sperm maturation, maintenance, and release, with multiple proteases acting to release sperm from the seminal clot in humans, as well as promotion of egg laying in insects [86–89]. Two serine proteases in the *An. gambiae* female reproductive tract have roles in normal processing of the mating plug and subsequent release of the hormone 20E, a regulator of post-mating responses in *An. gambiae* [90]. Importantly, proteases can facilitate amplification of a pathway via signaling cascades with sequential activation of multiple proteases by cleaving propeptides [86]. AAEL014567/Oviductin has two predicted serine protease domains. Oviductins in the oviduct of *Xenopus laevis* and *Bufo japanicus* cleave egg envelope glycoproteins to facilitate fertilization [91, 92].

#### Transcripts encoding proteins with predicted small molecule binding or hormone transport domains

AAEL001323 and AAEL006109/OBP23, which were similarly up-regulated in MAG extract-injected and mated females, have predicted functions in small molecule or hormone transport. AAEL001323 has a predicted hemolymph juvenile hormone-binding protein domain, and shares BLAST alignment with the takeout family of genes involved in metabolism, aging, and courtship of males [93]. The highest observed up-regulated transcript in response to both MAG extract injection and mating is AAEL006109/OBP23, a member of the odorant binding protein class [94]. OBP23 is expressed in a variety of tissues, such as male antennae and female rostrums [95, 96] and is up-regulated in the midgut after Zika infection in *Ae. aegypti* (Maria Onyango, personal communication). OBPs have been identified in the reproductive tracts of male and female insects (reviewed in [97]), including the moth *Athetis dissimilis* [98], and the seminal fluid of *Drosophila* [99] (reviewed in [100]). The role of odorant binding proteins in reproduction is not clear, but they might potentially act as carriers of pheromones transferred to females during mating.

#### Transcripts encoding gene-regulatory proteins

Transcription factors can stimulate or repress gene expression in pathways that are important for fertility, thus amplifying initial mating signals and potentially leading to post-mating changes. We identified two down-regulated genes with domains involved in gene expression regulation in both MAG extract-injected and mated LRTs. AAEL004097/Hairy & Enhancer of Split has a helix-loop-helix orange (HLH-O) domain. Proteins containing this domain are implicated in mosquito and *Drosophila* embryogenesis [101]. Knockdown of HLH-O domain genes in red flour beetle *Tribolium castaneum* revealed roles for these proteins in female survival, reproduction, and embryogenesis [102]. AAEL003861/BMP-induced factor has a BTB/POZ domain (Broad-complex, Tramtrack & bric-a-brac/poxvirus & Zn finger) which has characterized roles in development [103]. For example, the *Drosophila* BTB/POZ-containing protein MAMO, located in germ cells, activates factors required for completion of meiosis in order to successfully generate zygotes [104]. Additional analysis of the upstream genomic regions of SFP-responsive DEGs could reveal shared binding motifs for transcription factors regulating post-mating responses, but a larger sample of DEGs than we detected here would be needed to increase the power of the algorithms used to predict these sites.

#### Additional MAG extract-responsive transcripts

Other DEGs observed in LRTs from both MAG extract-injected and mated females have a variety of predicted functions. This includes up-regulation of AAEL019604, a gene that shares sequence alignment with a synaptic vesicle membrane protein, and down-regulation of AAEL014450, a peptidyl-glycine alpha-amidating monooxygenase which generates alpha-amidated peptides. Amidation of C-terminal amino acids is required for activity of hormones and neuropeptides. We previously reported the up-regulation of RNAs encoding transferrin after mating [46] and observed a similar increase in transcript levels after MAG extract injection. LRTs from MAG extract-injected and mated females had lower transcript levels of AAEL008342, which encodes a monocarboxylate transporter. Monocarboxylate transporters were also down-regulated in female honeybees injected with seminal fluid when compared to buffer-injected [105]. Down-regulation of oxidoreductase (AAEL09685) and up-regulation of AAEL012382, which contains a predicted C-terminal nucleosidase domain, and of AAEL014719, an inosine-uridine preferring nucleoside hydrolase, were also detected. Three down-regulated RNAs (AAEL023640, AAEL024292, AAEL022727) and one up-regulated RNA (AAEL026265) from both mating and MAG extract-injection are annotated as non-coding RNAs. The term non-coding RNA encompasses multiple species of RNA. The four ncRNAs we detected are all longer than 200 bp, consistent with what would be considered long noncoding (lncRNAs) (reviewed in [106]). Studies in vertebrates and invertebrates have demonstrated that lncRNAs have regulatory roles in gene expression in a variety of cellular and biological processes (reviewed in [106, 107]). Thus, the putative lncRNAs we identified could be interesting targets to explore for potential roles in modulating post-mating responses.

In summary, identification of common DEGs from both studies demonstrate that MAG extract injection can recapitulate some gene-expression changes in the LRT observed after mating.

### Comparison of DEGs from MAG extract-injected LRTs with those in spermathecae from mated females identify spermathecal transcript changes that are responsive to seminal fluid proteins

There are post-mating transcriptome changes in *Ae. aegypti* spermathecae, the sperm storage organs within the female LRT [57, 108]. Spermathecae store and maintain sperm, making this tissue vital for successful reproduction, particularly in monandrous mosquito species where stored sperm fertilize eggs for the remainder of a female’s life (up to 100 days or more [87]). We compared our MAG extract-injected LRT DEGs with genes recently reported in *Ae. aegypti* to be differentially expressed in the spermathecae of non-blood fed females at 24 h after mating [57] (Fig. 3B, C, Supplementary Tables 2, 3). We found 15 genes that overlapped between spermathecal tissue from mated females and LRTs from MAG extract-injected females (LRT includes the spermathecae). These included DEGs involved in immune pathways (AAEL019728), oxidative stress (AAEL009685), peptidase function (AAEL010867, AAEL014567, AAEL022646), neuronal function (AAEL014450, AAEL019604) and small molecule/hormone transport (AAEL001323, AAEL006109). These are consistent with protein classes identified in the spermathecae of *Ae. aegypti* after mating [57, 108], and also from transcriptional analysis of sperm storage organs after mating in Drosophila [109], *An. gambiae* [53], and ant queens [110]. Similar to what was found in the comparison of MAG extract-injected with mated LRTs (Supplementary Table 3), four of the overlapping genes (AAEL009291, AAEL000037, AAEL003832, AAEL003857) were also differentially expressed with saline injection and have functions consistent with immune pathways. Furthermore, 13 of the 15 overlapping genes, including all four that were also induced by saline, were identified as DEGs at 24 h in the mated LRT [46], and 14 of the 15 were similarly altered in the spermathecae of mated females 7 days post-eclosion [108], demonstrating consistency in our results. MAG extract-injected DEGs that overlap with transcript changes in the spermathecae after mating reflect responses that are likely induced by transferred SFPs.

## Conclusions

We have identified transcriptome responses in female *Ae. aegypti* reproductive tissues to seminal fluid protein-containing extracts that highlight important functional responses, including immunity, metabolism, and protease activity, that take place after mating. We focused on the role of seminal fluid proteins in this response, even though seminal fluid also contains small molecules, metabolites, and RNAs that could potentially also trigger post-mating responses in females, because our earlier work showed that the inducers of post-mating responses were heat-labile, suggesting that they are most likely proteinaceous [22, 29]. The genes identified as responsive to mating that are not responsive to MAG-extract injection could reflect genes that respond to other aspects of mating, such as receipt of sperm or physical contact. It is also possible that we were not able to capture the full extent of DEGs regulated by SFPs. The statistical power to detect smaller, yet significant, transcript changes was hampered by a lower-than-expected number of reads that mapped to genic regions of the *Ae. aegypti* reference genome assembly. Introduction of SFPs via intrathoracic injection into the hemocoel, rather than directly into the LRT as in the case of natural mating, could also contribute to an incomplete characterization of all relevant DEGs that respond to SFPs. The transcript-level changes identified in this analysis lay the groundwork for further characterization of female genes and associated pathways that are activated by SFPs provided by males. This study represents an important step in understanding the molecular basis of male-female mating interactions that are required for reproductive success and the promotion of post-mating responses elicited by SFPs. These findings provide crucial potential targets for the creation of novel vector control strategies such as ones that target these pathways to disrupt successful reproduction, ultimately reducing vector populations.

## Methods

### Mosquitoes

We used a Thai wild-type strain of *Ae. aegypti*, established in 2011 and supplemented with field collected mosquitoes from Thailand 1 year prior to our experiments. Mosquitoes were reared as described previously [29].

### Preparation and injection of seminal fluid proteins

Males were anesthetized on ice and their accessory glands (MAG) with seminal vesicles (hereafter referred to as MAG extract) were dissected from 100 five- to six-day-old male adult *Ae. aegypti* in modified PBS (137 mM NaCl, 2.7 mM KCl, 10 mM Na_2_HPO_4_, 3 mM KH_2_PO_4_, 2 mM CaCl_2_ pH 7.0) in a ratio of 1 accessory gland pair per microliter of buffer. Tissue was homogenized with a motorized pestle for ten 1 s pulses, followed by sonication in a BIORUPTOR (Diagenode, Denville, NJ) at 4 °C on “high” setting with a cycle of 15 s on, 15 s rest, 15 s on. After centrifugation at 25,000 g for 30 min at 4 °C, the supernatant was recovered and snapfrozen in liquid N_2_ prior to storage at − 80 °C. All MAG extracts had similar protein concentrations (1.17 μg/μl, 1.07 μg/μl, 1.05 μg/μl) as determined with Micro BCA kit (Thermo Fisher Scientific, Waltham, MA). From experiments in which we measured, separately, the protein content of MAGs and seminal vesicles, we estimate that the protein contribution from seminal vesicle was ~ 5% of the total lysate. To rule out any contribution of sperm to our results, we stained the extracts with DAPI, to count the number of sperm-heads present. We found only negligible amounts of sperm, comprising < 1 sperm potentially injected per female. Biological activity in inducing post-mating responses was verified for the MAG extract by injecting them into a subset of virgin females, exposing those females to males 2 days later to provide an opportunity for mating overnight, and dissecting their spermathecae to assess whether or not sperm were present; the latter outcome verified that the extract was active, as it prevented the females from mating. Similarly treated saline-injected females all mated with the males provided.

Virgin females (4 to 5 days post-eclosion) were injected intrathoracically with 0.25 μl of modified PBS or MAG extract using a Nanoject III (Drummond Scientific Co., Broomall, PA). The amount of injected MAG extract represents 0.25 male accessory gland equivalents, approximately the amount transferred to females during a natural mating [25, 111]. At 24 h post-injection, 26 lower reproductive tracts (bursa, spermathecae, and oviducts) were dissected from females in each injection group in chilled PBS and placed immediately into 150 μl Trizol (Thermo Fisher Scientific, Waltham, MA). Non-injected virgin females (*n* = 26) were dissected as controls (Fig. 1A). All treatments were performed and analyzed in three biological replicates (each with 26 individuals pooled).

### RNA isolation and library construction

Total RNA was extracted with 500 μl total Trizol following modifications to manufacturer’s protocol with utilization of Phaselock gel tubes and GlycoBlue carrier (Thermo Fisher Scientific, Waltham, MA). Following resuspension of the pellet in RNAse-free water, RNA was quantified using a Qubit Broad Range RNA Kit (Thermo Fisher Scientific, Waltham, MA) and quality assessed on an Agilent BioAnalyzer 2100 (Agilent Technologies, Santa Clara, CA). Total RNA (250 ng) was used to prepare libraries using the QuantSeq 3’mRNA library (REV) kit (Lexogen, Greenland, NH). Final libraries were quantified with a Qubit High Sensitivity DNA kit (Thermo Fisher Scientific, Waltham, MA) and quality was assessed on an Agilent BioAnalyzer 2100 (Agilent Technologies, Santa Clara, CA). Sequencing was performed at the Cornell Biotechnology Resource Center on a NextSeq500 with the custom sequencing primer (CSP) version 5 supplied by Lexogen (75 bp paired-end reads).

### Read processing, alignment, and differential expression analysis

After checking read quality with FastQC, paired-end reads were aligned to the *Ae. aegypti* genome (version AaegL5) with Hisat2 using default parameters [112]. In addition to the samples generated in this study, we included in our analysis post-mating RNA-seq samples that were generated in our previous study [46] (Supplementary Fig. 1). Alignments were evaluated with Qualimap [113, 114] and gene counts were extracted using the AaegL5 exon annotations. We detected reads in all replicates and treatments that mapped to intergenic and intronic regions (~ 30–40%) (Supplementary Fig. 3A, 3B). In addition, reads from one of the three non-injected control group library were excluded from the analysis due to a lower alignment rate (~ 20% lower than other samples) and higher read pair discordance (3% higher than other samples) (Supplementary Fig. 3C, 3D). Additionally, replicate analysis before RUVSeq batch correction found this sample to be an outlier with respect to the grouping of the other samples and expression profile Pearson correlation coefficients (Supplementary Fig. 3E, 3F).

The gene counts matrix was filtered to remove genes with low-abundance RNAs (CPM < 5). For differential abundance analysis, we controlled for sources of erroneous variation using RUVseq with *k* = 3 [115] then fit a quasi-likelihood generalized linear model that incorporates the RUVseq residuals to the count data using edgeR [116] (Supplementary Fig. 4). Replicates grouped together as determined via multi-dimensional scaling (Fig. 1B). The differential expression analysis involved setting up the following contrasts at 24 h post-injection: (1) between the saline-injected and non-injected samples, (2) between the MAG extract-injected and non-injected samples, and (3) between MAG extract-injected and saline-injected samples (Supplementary Fig. 5). We defined genes as significantly differentially expressed between treatments if transcript abundance was > 2-fold with a Benjamini-Hochberg corrected false-discovery rate (FDR) < 0.05 [117]. Identified DEGs from the comparison of MAG extract-injected and non-injected samples were further compared to DEGs identified in the LRT or spermathecae 24 h after mating [46, 57]. Functional annotation resources were acquired from VectorBase (vectorbase.org) and were generated previously [16]. Gene Ontology (GO) enrichment analysis was carried out using GOSeq [118]. For GeneIDs not characterized in VectorBase, we searched for predicted conserved domains with NCBI Conserved Domain database [119] or similar genes by tBLASTn [120].

## Supplementary Information

**Additional file 1.** Supplementary Tables 1-3 and Figures 1-5.

**Additional file 2.** Supplementary File 1-Mated vs virgin updated analysis.

## Data Availability

The Illumina sequence reads are available through the Sequence Read Archive (SRA) under project accession PRJNA701279. The processed data files and analysis scripts are available through a GitHub repository: https://github.com/YazBraimah/Aegypti_MAG_Saline_RNAseq.
