# Supplementary material for: Seminal fluid proteins induce transcriptome changes in the Aedes aegypti female lower reproductive tract
Source: BMC Genomics. 2021 Dec 15;22:896. doi: 10.1186/s12864-021-08201-0 (PMC8672594; doi:10.1186/s12864-021-08201-0)
Supplement: Supplementary file 1 — Additional file 1. Supplementary Tables 1-3 and Figures 1-5. [file 12864_2021_8201_MOESM1_ESM.pdf]

SUPPLEMENTARY INFORMATION

Seminal fluid proteins induce transcriptome changes in the *Aedes aegypti* female lower reproductive tract

I. Alexandra Amaro[1], Yasir H. Ahmed-Braimah[2], Garrett P. League[1], Sylvie A. Pitcher[1], Frank W. Avila[3], Priscilla C. Cruz[1], Laura C. Harrington[1*], and Mariana F. Wolfner[4*]

1 - Department of Entomology, Cornell University, Ithaca, NY 14853, United States,

2 - Department of Biology, Syracuse University, Syracuse NY 13244, United States,

3 - Max Planck Tandem Group in Mosquito Reproductive Biology, Universidad de Antioquia, Medellín 050010, Colombia

4 - Department of Molecular Biology and Genetics, Cornell University, Ithaca, NY 14853, United States

Supplementary Table 1. Transcript-level changes (10 down-regulated, 122 up-regulated) and their respective fold change (logFC) in the virgin female LRT 24 h after saline injection compared to non-injected females. The logFC for the 42 genes that are altered after injection of MAG extract are included. NA=not annotated

GeneID	VectorBase Description	Saline-injected vs non-injected logFC	MAG extract-injected vs non-injected logFC
AAEL024784	NA	-2.76	
AAEL014171	NA	-2.57	-3.97
AAEL000547	pickpocket	-1.82	
AAEL013320	translocon-associated protein delta subunit	-1.77	
AAEL024471	NA	-1.66	
AAEL005032	NA	-1.62	
AAEL026602	NA	-1.39	-1.72
AAEL025498	NA	-1.33	
AAEL005528	NA	-1.14	
AAEL001766	leucine-rich transmembrane proteins	-1.04	
AAEL002080	septin interacting protein, putative	1.00	
AAEL002124 (CLIPD6)	Clip-Domain Serine Protease family D	1.00	1.11
AAEL003883	NA	1.02	
AAEL005127	ribonuclease UK114, putative	1.02	
AAEL020823	NA	1.02	
AAEL002686	testisin precursor, putative	1.04	1.23
AAEL013844	diazepam binding inhibitor, putative	1.06	
AAEL024669	NA	1.07	1.71
AAEL004120	Niemann-Pick Type C-2, putative	1.08	1.39
AAEL004247	Sialin, Sodium/sialic acid cotransporter, putative	1.09	
AAEL012993	NA	1.11	
AAEL008303	calponin/transgelin	1.12	1.17
AAEL010029	NA	1.13	
AAEL015493	NA	1.13	1.23
AAEL008936	NA	1.14	1.76
AAEL009291	retinoid-inducible serine carboxypeptidase (serine carboxypeptidase)	1.14	1.44
AAEL012725	NA	1.15	
AAEL024161	NA	1.17	
AAEL017345	NA	1.17	1.66
AAEL001887	glutamine synthetase 1, 2 (glutamate-amonia ligase) (gs)	1.18	1.04
AAEL000037 (CLIPB35)	Clip-Domain Serine Protease family B.	1.18	1.63
AAEL021625	NA	1.74	
AAEL017536 (GRRP)	holotricin glycine-rich repeat protein (GRRP) anti-microbial peptide	1.75	2.06
AAEL004976	NA	1.78	
AAEL023353	NA	1.81	2.23
AAEL005748	elastase, putative	1.82	2.07
AAEL002557	cationic amino acid transporter	1.84	
AAEL009948 (ALDH9948)	aldehyde dehydrogenase	1.87	
AAEL010094	cyclin b	1.91	
AAEL026440	NA	1.95	
AAEL018212	Ubiquitinyl hydrolase 1	2.00	
AAEL004809	NA	2.00	2.86
AAEL024914	Putative synaptic vesicle transporter svop	2.00	
AAEL017144	NA	2.01	2.52
AAEL008753	NA	2.02	2.70
AAEL020097	NA	2.03	2.89
AAEL002624	serine protease	2.04	2.38
AAEL023625	NA	2.08	
AAEL003832 (DEFC)	defensin anti-microbial peptide	2.10	3.53
AAEL015052	NA	2.13	
AAEL008580	NA	2.13	
AAEL008510	sphingosine kinase a, b	2.14	
AAEL008473	cysteine-rich venom protein, putative	2.14	2.82
AAEL004172	tubulin alpha chain	2.17	
AAEL000604	NA	2.19	
AAEL009773	geminin, putative	2.20	
AAEL010773 (CLIPE10)	Clip-Domain Serine Protease family E. Protease homologue.	2.22	2.75
AAEL018241	NA	2.24	2.93
AAEL000776	NA	2.24	
AAEL003404	NA	2.25	
AAEL008137	NA	2.34	
AAEL007237	NA	2.34	

GeneID	VectorBase Description	Saline-injected vs non-injected logFC	MAG extract-injected vs non-injected logFC	GeneID	VectorBase Description	Saline-injected vs non-injected logFC	MAG extract-injected vs non-injected logFC
AAEL009959	pre-mRNA splicing factor prp8	1.19		AAEL006208	NA	2.35	
AAEL017098	NA	1.21		AAEL002184	F-actin capping protein beta subunit	2.36	
AAEL007102	trypsin, putative	1.21		AAEL004462	NA	2.45	
AAEL003483	NA	1.22	1.95	AAEL023532	NA	2.51	
AAEL006576	clip-domain serine protease, putative	1.22	1.25	AAEL011532	NA	2.56	
AAEL021092	NA	1.23		AAEL026038	NA	2.63	
AAEL009476	NA	1.31	1.27	AAEL006726 (inx4)	Innexin	2.64	
AAEL028105	NA	1.32		AAEL021169	NA	2.81	
AAEL003469	NHP2 protein, putative	1.32		AAEL024562	NA	2.98	
AAEL019870	NA	1.33	1.39	AAEL002586	NA	3.00	
AAEL004176	microtubule binding protein, putative	1.33		AAEL006971	NA	3.02	5.12
AAEL010970	NA	1.35		AAEL005049	heterogeneous nuclear ribonucleoprotein	3.03	
AAEL002585	serine protease	1.38	2.10	AAEL003857 (DEFD)	defensin anti-microbial peptide	3.03	4.03
AAEL000636	NA	1.38		AAEL001666	NA	3.07	
AAEL011408 (CTL21)	C-Type Lectin (CTL21)	1.40	1.44	AAEL000961	NA	3.08	
AAEL021077	NA	1.40		AAEL007783	centromere protein-A, putative	3.09	
AAEL015533	NA	1.40	1.56	AAEL026714	NA	3.11	
AAEL000786	NA	1.41	1.63	AAEL026111	NA	3.12	
AAEL024387	Putative serine protease	1.43		AAEL020191	NA	3.20	
AAEL002822 (Arp5)	actin-related protein 5	1.46		AAEL013813	NA	3.21	
AAEL015432	trypsin, putative	1.49		AAEL007657	low-density lipoprotein receptor (ldl)	3.31	
AAEL013142	NA	1.49		AAEL006492	centrin, putative	3.37	
AAEL002235	NA	1.53		AAEL007097	4-nitrophenylphosphatase	3.37	
AAEL000064	dopachrome-conversion enzyme (DCE) isoenzyme, putative	1.57	1.75	AAEL013338	lethal(2)essential for life protein, l2efl	3.47	
AAEL003299	NA	1.58	1.72	AAEL013300	NA	3.64	
AAEL026519	NA	1.61	1.88	AAEL008829	NA	3.69	
AAEL003657	zinc finger protein	1.61		AAEL010097	NA	3.91	
AAEL004978	DEAD box ATP-dependent RNA helicase	1.62		AAEL000335	lamin	4.05	
AAEL021666	NA	1.65	1.49	AAEL004316	NA	4.11	
AAEL002652	NA	1.65		AAEL004388 (HPX8A)	heme peroxidase	4.41	
AAEL011424	Histone H3	1.71		AAEL019534	NA	4.51	
AAEL000800	microsomal dipeptidase	1.72		AAEL007370	NA	5.66	5.67
AAEL017132 (LYSC4)	C-Type Lysozyme (Lys-C).	1.73	1.78	AAEL027148	NA	6.31	
AAEL007371	NA	1.73	2.56	AAEL004386 (pxt)	chorion peroxidase	6.69	
AAEL003903	acid phosphatase-1	1.73		AAEL008098 (PIWI2)	PIWI	7.88	

Supplementary Table 2. DEGs and their respective fold change (logFC) at 24 h post MAG extract injection compared to non-injected females. DEGs that are also altered due to saline injection compared to non-injected females are not included here, and rather are listed in table S1. The logFC of DEGs that are significantly different between MAG extract-injected and saline-injected females are listed. The logFC values for those genes that are also observed to have significant transcript-level changes in the LRT [1] and spermathecae (SP) [2] from mated females compared to virgins from previous studies are included. For GeneIDs that are undefined in VectorBase and select additional GeneIDs, information from NCBI Conserved Domain Search and BLAST (tBLASTn) is noted. NA=not annotated.

GeneID	VectorBase Description	Predicted Domain (PD) / BLAST (B)	LRT – MAG extract-injected vs non-injected	LRT – MAG extract-injected vs saline-injected	LRT – Mated vs Virgin	SP – Mated vs Virgin
**IMMUNE FUNCTION**						
AAEL000200	NA	VWD domain (PD)	1.00		1.30	
AAEL019650	NA	Nimrod (B)	1.01	1.14		
AAEL019728 (SOCS)	Suppressor of Cytokine Signaling		1.01		2.80	3.52
AAEL001414 (LRIM9)	leucine-rich immune protein (Short)		1.14			
AAEL000543 (CTLMA11)	C-Type Lectin (CTLMA11) - mannose binding.		1.15			
AAEL001794	macroglobulin/complement		1.41		1.00	
AAEL009474 (PGRPS1)	Peptidoglycan Recognition Protein (Short)		1.53			
AAEL019745	NA	Peptidoglycan recognition protein (PGRP) (PD)	1.80			
AAEL017023	NA	Macroglobulin (PD) / CD109 (B)	1.80			
AAEL014382 (CTLMA14)	C-Type Lectin (CTL) - mannose binding.		1.82			
AAEL020192	NA	Fibrinogen related domain (PD)	1.86		2.20	
AAEL007626 (GNBPA1)	Gram-Negative Binding Protein (GNBP) or Beta-1 3-Glucan Binding Protein (BGBP).		2.19		1.90	
AAEL003723 (LYSC11)	C-Type Lysozyme (Lys-A).		2.22			
AAEL012353 (CTL15)	C-Type Lectin (CTL).		2.46			
AAEL029038	NA	Cecropin (PD/B)	2.53			
AAEL006355 (SCRC1)	Class C Scavenger Receptor (Sushi/SCR/CCP MAM and Somatomedin B domains).		3.76			
AAEL029044 (CECE)	Cecropin		3.82	1.90	3.90	
**PROTEASE FUNCTION**						
AAEL001178	serine protease	Trypsin-like serine protease (PD)	-1.15		-3.30	
AAEL020362	NA	Trypsin-like serine protease (PD)	1.07			
AAEL010867	serine protease	Trypsin-like serine protease (PD)	1.26		2.90	1.97
AAEL002301	serine protease	Trypsin-like serine protease & Protease CLIP Domain (PD)	1.35			
AAEL002580	NA	Trypsin-like serine protease & Protease CLIP Domain (PD)	1.35			
AAEL014567	oviductin	2 Trypsin-like serine protease Domains (PD)	1.59	1.17	2.99	2.72

GeneID	VectorBase Description	Predicted Domain (PD) / BLAST (B)	LRT – MAG extract-injected vs non-injected	LRT – MAG extract-injected vs saline-injected	LRT – Mated vs Virgin	SP – Mated vs Virgin
**PROTEASE FUNCTION (Continued)**						
AAEL005431 (CLIPB37)	Clip-Domain Serine Protease family B.	Trypsin-like serine protease & Regulatory CLIP Domain (PD)	1.86			
AAEL002610	serine protease	Trypsin-like serine protease & Protease CLIP Domain (PD)	1.86			
AAEL005792 (CLIPE8)	Clip-Domain Serine Protease family E. Protease homologue.	2 Trypsin-like serine protease Domains & CLIP/disulfide knot Domain (PD)	2.23			
AAEL005093 (CLIPB46)	Clip-Domain Serine Protease family B.	Trypsin-like serine protease & Regulatory CLIP Domain (PD)	2.44			
AAEL022646	Larval chymotrypsin-like protein	Trypsin-like serine protease (PD)	3.89			-7.30
**SMALL MOLECULE/HORMONE TRANSPORT**						
AAEL001323	NA	Haemolymph juvenile hormone-binding protein (JHBP) (PB) / takeout (B)	1.10	1.03	4.90	3.44
AAEL001964	protein serine/threonine kinase, putative	Insect pheromone-binding family (PB) / Ejaculatory bulb-specific protein 3 (B)	1.63	1.35		
AAEL009927	NA	Haemolymph juvenile hormone-binding protein (JHBP) (PB)	1.99			
AAEL006109 (OBP23)	odorant binding protein OBP23		4.48		6.80	8.57
**NEURONAL FUNCTION**						
AAEL014450	Peptidyl-glycine alpha-amidating monooxygenase	NHL/PAL-like domain (PD)	-1.11		-2.20	-1.80
AAEL019604	NA	MDR3 Domain (PD) / Synaptic vesicle membrane protein VAT-1 homolog-like (B)	1.06		1.30	2.74
AAEL003413	F-spondin		1.12			
AAEL023634	NA	Neurofilament light polypeptide-like (B)	1.55			
AAEL005945 (GPRDOP4)	GPCR Dopamine Family		1.60	1.24		
**TRANSCRIPTION CONTROL**						
AAEL004097	enhancer of split protein, putative	Orange & basic helix-loop-helix-orange (bHLH-O) domain (PD)	-1.23		-2.00	
AAEL003861	bmp-induced factor	BTB (Broad-Complex, Tramtrack and Bric a brac) /POZ (poxvirus and zinc finger) domain (PD)	-1.00		-1.40	
AAEL013321	NA	Zn Finger (PD)	1.77	1.64		
**TRANSPORT AND METABOLISM**						
AAEL008342	monocarboxylate transporter		-1.48		-1.10	
AAEL009232	long-chain-fatty-acid coA ligase		-1.29			
AAEL009685	oxidoreductase		-1.07		-4.80	-10.94
AAEL001194 (FAS1)	fatty acid synthase		-1.02			
AAEL014248	NA	Riboflavin transporter (B)	1.05			
AAEL015304	NA	Ester hydrolase C11orf54 homolog (B)	1.10			
AAEL015458 (Tf1)	transferrin		1.14		2.70	
AAEL009129 (CYP6Z9)	cytochrome P450		1.14			
AAEL014719	inosine-uridine preferring nucleoside hydrolase		1.35		1.10	

GeneID	VectorBase Description	Predicted Domain (PD) / BLAST (B)	LRT – MAG extract-injected vs non-injected	LRT – MAG extract-injected vs saline-injected	LRT – Mated vs Virgin	SP – Mated vs Virgin
**TRANSPORT AND METABOLISM (Continued)**						
AAEL012382	NA	C-terminal nucleotidase & MPP (CD73 ecto-5'-nucleotidase and related proteins, N-terminal metallophosphatase) domain (PD) / 5UNC-like (B)	1.38		3.50	4.69
AAEL006002	NA	Major facilitator superfamily domain-containing protein 1 (PD)	1.48			
AAEL000271	gamma-glutamyl hydrolase		1.54			
AAEL017455	NA	ribonuclease E Domain (PD)	1.55			
AAEL014258	monocarboxylate transporter		2.22			
AAEL004025	glucose dehydrogenase		2.39			
**ADDITIONAL GENES**						
AAEL025361	NA - Non-Coding RNA		-2.52			
AAEL022704	NA		-1.96			
AAEL004570	NA	Zn-finger associated domain (PD)	-1.68			
AAEL028121	NA - Non-Coding RNA		-1.64			
AAEL023640	NA - Non-Coding RNA		-1.51		-5.40	
AAEL024292	NA - Non-Coding RNA		-1.51		-1.30	
AAEL000240	NA	ubiquinone biosynthesis protein COQ9 (B)	-1.24			
AAEL025444	NA - Non-Coding RNA		-1.19			
AAEL025362	60S ribosomal protein L10		-1.10			
AAEL022727	NA - Non-Coding RNA		-1.07		-3.60	
AAEL011478	cytoplasmic dynein light chain		-1.06			
AAEL014843	heat shock protein		1.01			
AAEL024482	NA		1.03			
AAEL001901	MRAS2, putative	P-loop containing Nucleoside Triphosphate Hydrolases (PD) / ras-related and estrogen-regulated growth inhibitor-like protein (B)	1.07			
AAEL001851	NA	Chromosome segregation ATPase & gp58-like protein (PD)	1.10			
AAEL017113	H/ACA ribonucleoprotein complex subunit		1.11			
AAEL023691	NA - Non-Coding RNA		1.16			
AAEL026403	NA - Non-Coding RNA		1.19			
AAEL026833	NA		1.20			
AAEL007778	leucine-rich transmembrane protein		1.41			
AAEL013262	NA	Glycoside hydrolase family 19, chitinase domain (PD) / endochitinase (B)	1.51			
AAEL022454	NA - Non-Coding RNA		1.53			
AAEL021795	NA		1.55			-1.66
AAEL012853	NA		2.00	1.00		
AAEL012856	NA		2.06		2.20	
AAEL029107	NA		2.40			
AAEL026265	NA - Non-Coding RNA		2.44		2.60	
AAEL025126	NA		2.47			
AAEL017380	NA		3.18	2.19		
AAEL001392	NA		3.52	2.00		
AAEL013515	pupal cuticle protein, putative		4.18	3.58		
AAEL008050	NA	Ribosome inactivating protein (RIP) (PD)	4.23			

Supplementary Table 3. Genes with differential gene expression between both MAG extract-injected and saline-injected compared to non-injected females, which also are differentially expressed in either the LRT [1] or Spermathecae (SP) [2] in mated females after 24 h. The degree of transcript change (logFC) is noted as is additional information from NCBI tools Conserved Domain Search and BLAST (tBLASTn) for select gene IDs. NA=not annotated.

GeneID	VectorBase	Predicted Domain (PD) / BLAST (B)	LRT – Saline-injected vs non-injected	LRT – MAG extract-injected vs non-injected	LRT – Mated vs. Virgin	SP – Mated vs. Virgin
AAEL026602	NA	L-ascorbate oxidase (PD) / Lacasse (B)	-1.39	-1.72	-1.31	
AAEL009291	retinoid-inducible serine carboxypeptidase	Serine Carboxypeptidase (PD)	1.14	1.44	1.21	2.20
AAEL015533	NA	2 Trypsin-like serine protease domains (PD)	1.40	1.56	1.07	
AAEL000037 (CLIPB35)	Clip-Domain Serine Protease family B.	Trypsin-like serine protease & Protease CLIP Domain (PD)	1.18	1.63	1.64	1.66
AAEL003832 (DEFC)	defensin anti-microbial peptide		2.10	3.53	2.81	4.10
AAEL003857 (DEFD)	defensin anti-microbial peptide		3.03	4.03	3.56	3.37

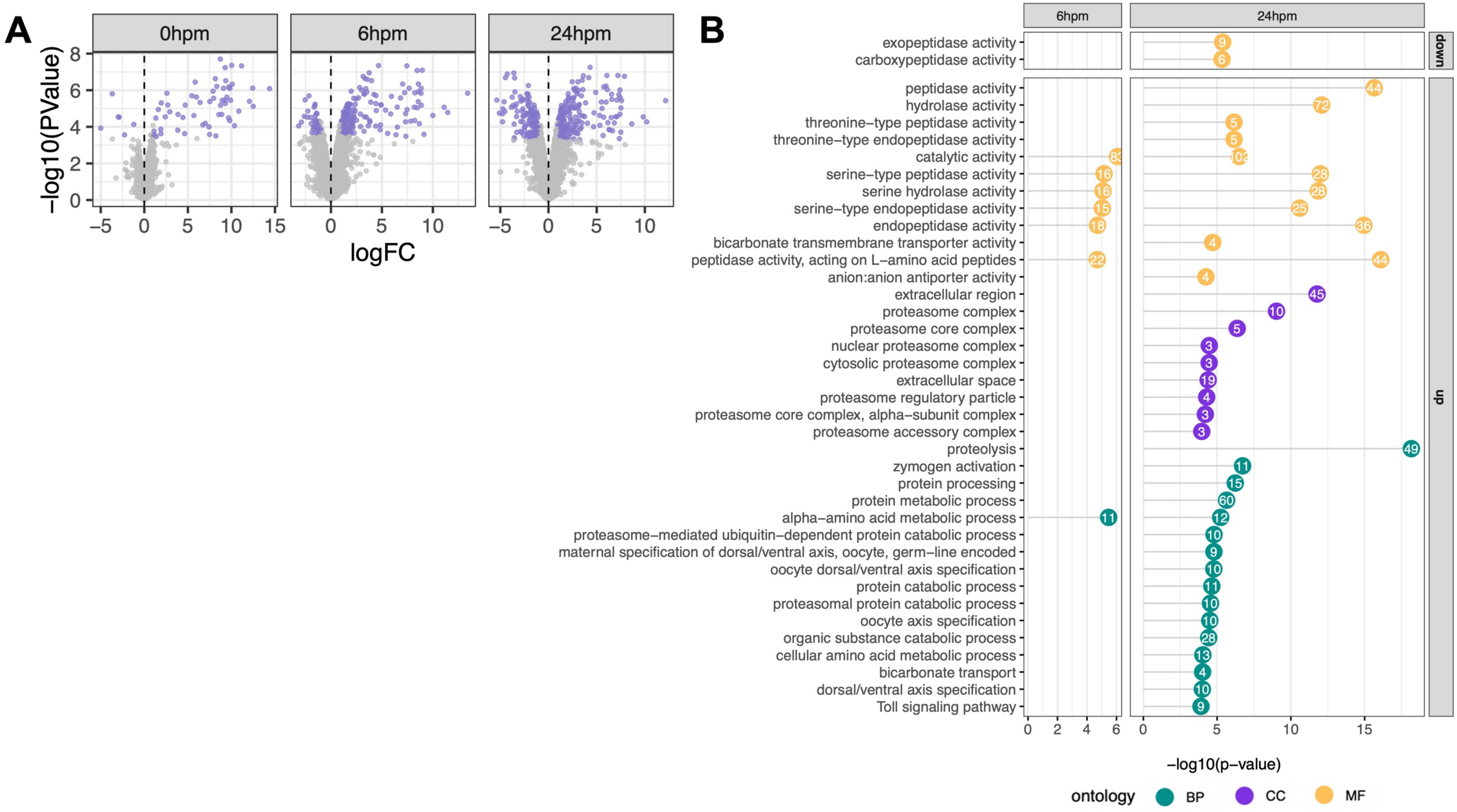

Supplementary Figure 1. Re-analysis of transcript-level changes in LRT of mated females [1]. (A) Volcano plots depicting all DEGs in the LRT between virgin and mated females at 0, 6, and 24 h post-mating (hpm). Significant transcript level changes highlighted in purple. (B) Enriched Gene Ontology (GO) Terms at 6 and 24 hpm. Circle denotes number of genes included in a particular GO term. BP=Biological Process; CC=Cellular Component; MF=Molecular Function.

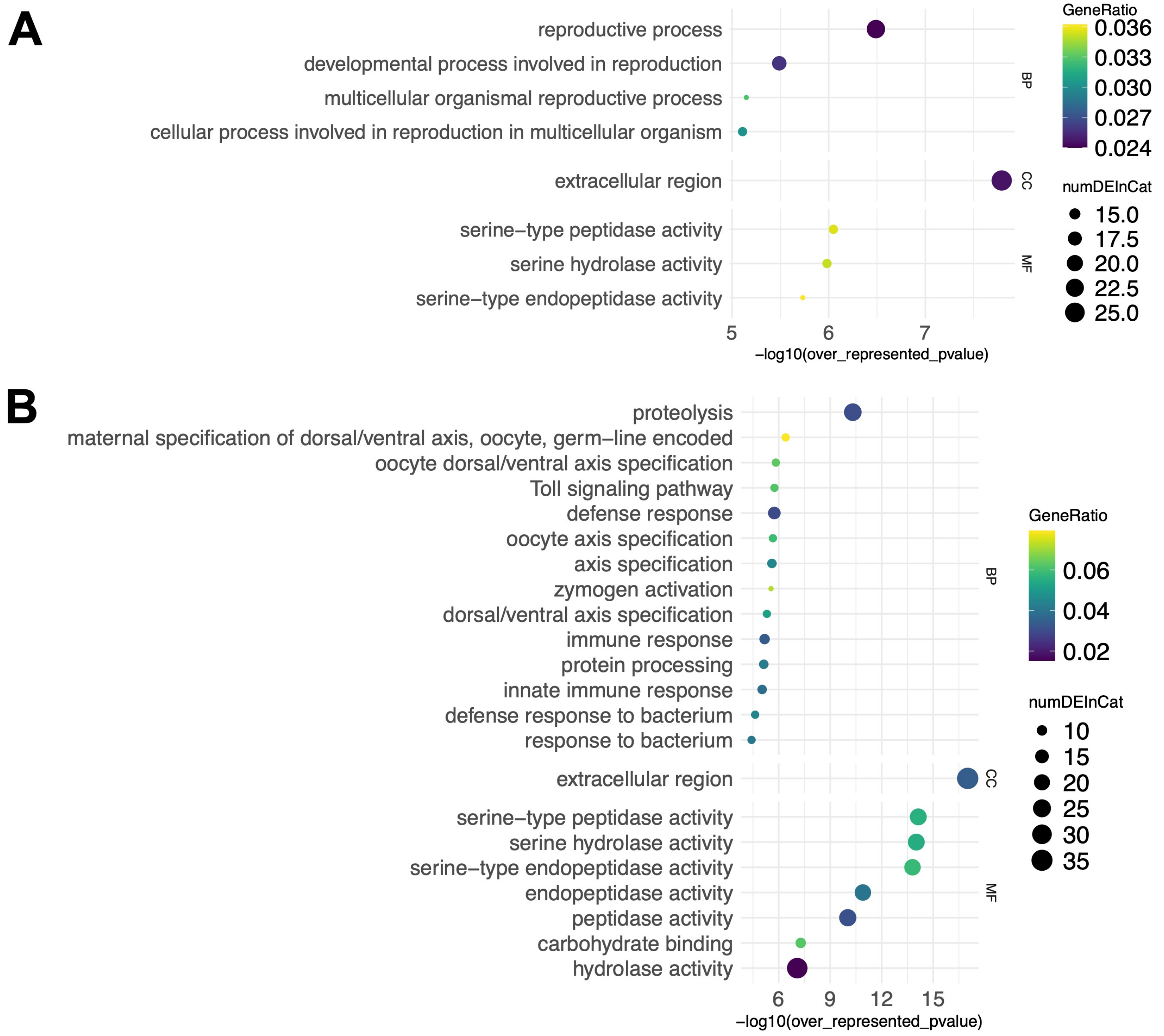

Supplementary Figure 2. Gene Ontology (GO) enrichment analysis. Enriched Gene Ontology (GO) Terms from DEGs at 24 h after injection with (A) saline or (B) MAG extract. BP=Biological Process; CC=Cellular Component; MF=Molecular Function.

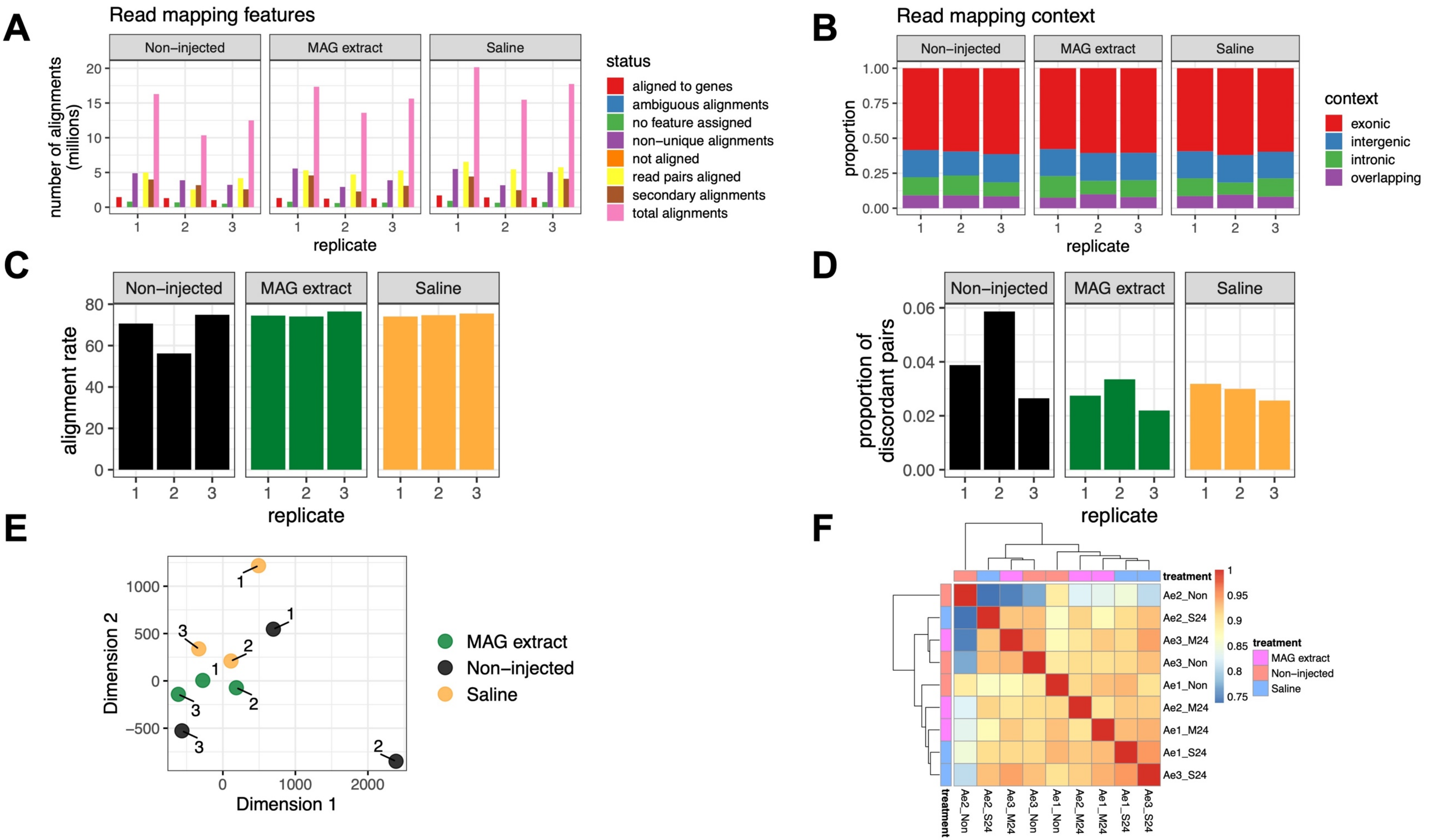

Supplementary Figure 3. Quality control of library sequencing reads. (A) Counts of read mapping features for all libraries from all replicates and treatments indicated by raw number. (B) Proportions of reads mapping to genomic features across all samples. (C) Alignment rate and (D) proportion of discordant pairs across all samples. Note that reads from the library for replicate 2 of non-injected females has a lower alignment rate (56.2% vs. 71-76% for other samples) and higher proportion of discordant pairs (6% vs. 2-3% for other samples). (E) Multi-dimensional scaling (MDS) plot and (F) Pearson correlation heatmap of all replicates and treatments prior to RUVSeq batch correction. Note that replicate 2 for non-injected treatment is outside the grouping of all other samples; hence this example was excluded from analyses, as noted in the text.

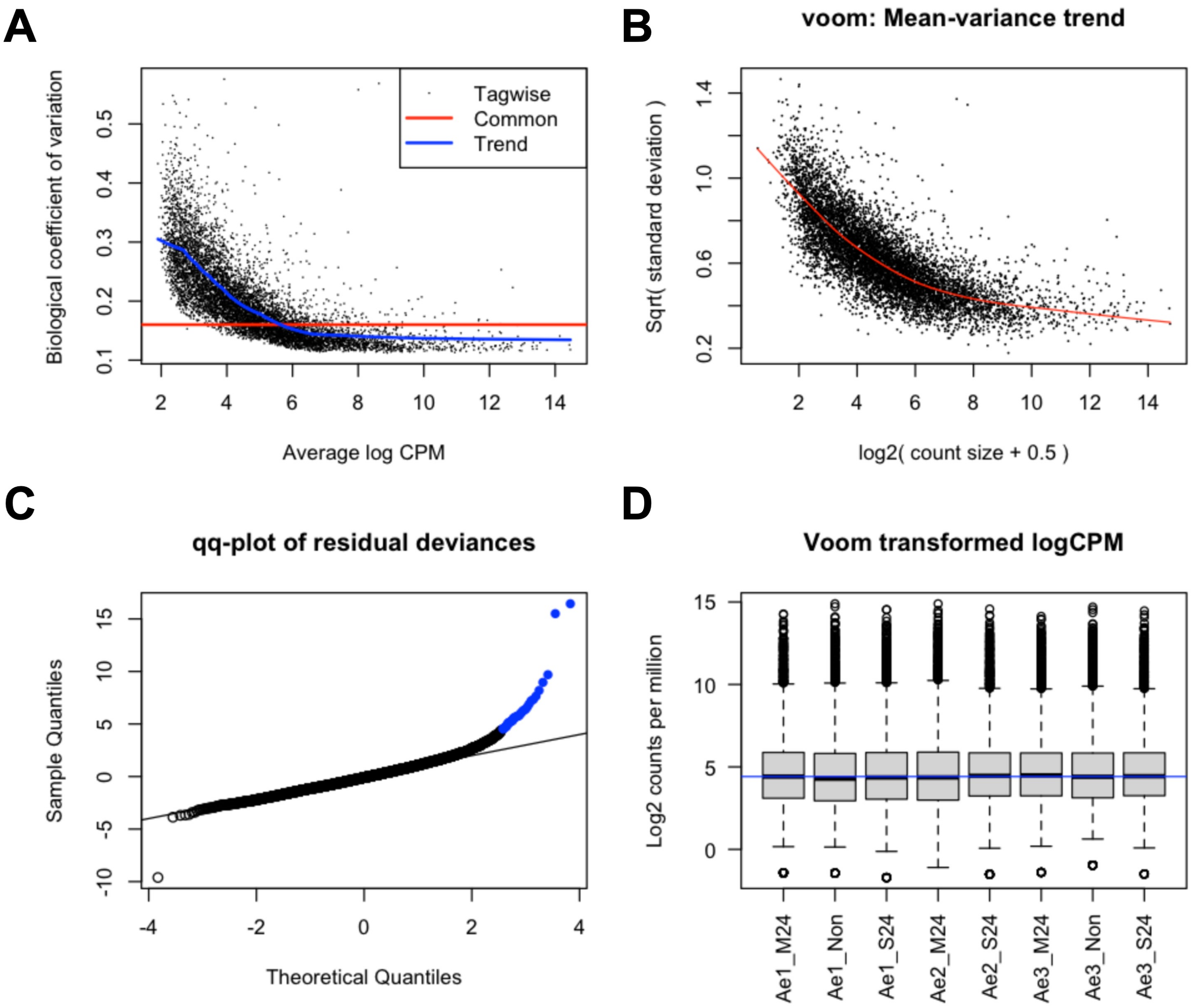

Supplementary Figure 4. Transcript normalization before differential expression analysis (A) Biological Coefficient Variation (BCV) mean-variance trend and model fit. (B) Voom precision weights for mean variance trends. (C) Quantile-Quantile (Q-Q plot) for the distribution of the sample transcript expression. (D) Log2 counts per million trends per sample using voom transformed log counts per million (logCPM).

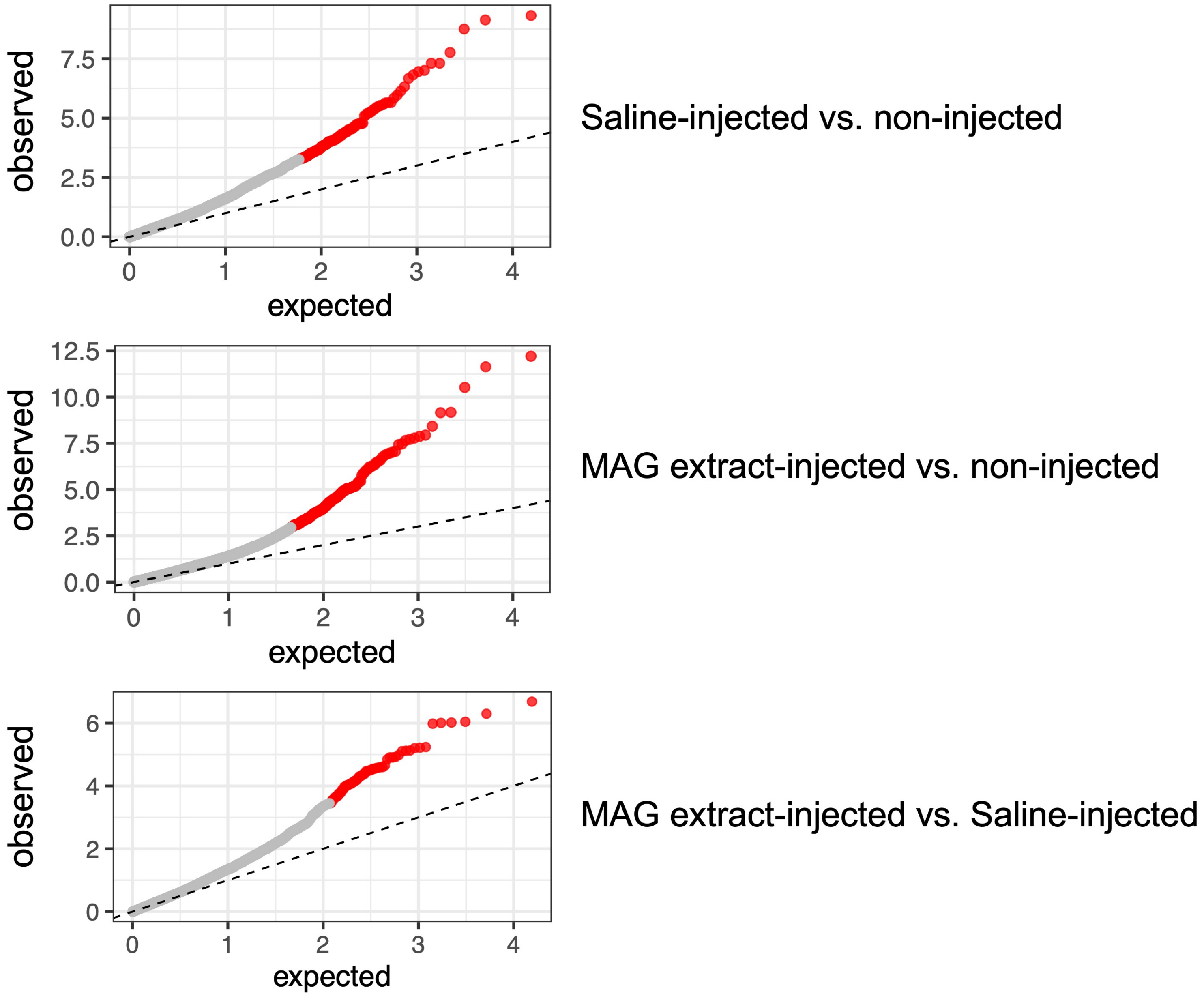

Supplementary Figure 5. Quantile-Quantile (Q-Q plot) for the distribution of all comparisons made in our analysis. The red dots show the genes that cross the 95% confidence interval to designate the differentially expressed genes. The dashed line shows the 95% normal distribution confidence intervals.

Supplementary References

1. Alfonso-Parra C, Ahmed-Braimah YH, Degner EC, Avila FW, Villarreal SM, Pleiss JA, et al. Mating-Induced Transcriptome Changes in the Reproductive Tract of Female *Aedes aegypti*. Benedict MQ, editor. PLoS Negl Trop Dis. Public Library of Science; 2016;10:e0004451.

2. Camargo C, Ahmed-Braimah YH, Amaro IA, Harrington LC, Wolfner MF, Avila FW. Mating and blood-feeding induce transcriptome changes in the spermathecae of the yellow fever mosquito *Aedes aegypti*. Sci Rep. 2nd ed. Nature Publishing Group; 2020;10:14899–13.
